# A Ministry of Health

**Published:** 1917-04-21

**Authors:** 


### A MINISTRY OF HEALTH.

### The Next Great Reform.

A recent deputation to the Local Government Board was historic, inasmuch as it indicated that the next great reform will be the creation of a Ministry of Health—a Department likely to be established at the Local Government Board, which now supervises most of the national health services.

The deputation was from all the Insurance Committees, and it had many objects; the first was to urge for the new Ministry of Health, the second to emphasise that housing must be the basis of health reform, and the third to ascertain the likely future of Insurance Committees in any scheme for the reorganisation of national health administration.

The deputation was introduced by Mr. Glyn Jones and received by Lord Rhondda, the President of the Local Government Board, and Mr. Hayes Fisher, the Parliamentary Secretary of that Department. In the course of speeches and in statements handed in the deputation laid most stress on the question of housing in connection with tuberculosis and sanatorium benefit under the Insurance Acts. The deputation further urged that power should be given to compel persons affected to enter sanatoria if necessary. Owing to the multiplicity of authorities, co-ordination is essential.

At the present time the following authorities at least are concerned with public health:—County Councils, local sanitary authorities (that is, borough, urban, and rural councils), Education Committees, Insurance Committees, Boards of Guardians, and factory inspectors. In addition to these there are approved societies, private employers, and insurance companies dealing with compensation for accidents. It will probably be difficult to amalgamate all the various health authorities, but the deputation feels strongly that if all the efforts of these bodies could be subject to one central health authority, enormous benefit would accrue.

The deputation had noticed that in two cases recently—for example, in the circulars issued by the Local Government Board in connection with schemes for maternity and child welfare, and also with schemes for the treatment of venereal diseases, Insurance Committees were omitted. The deputation drew attention to this fact in order to emphasise the necessity for the co-ordination of public health administration. All these matters, which are of urgent importance to the life of the nation, should be considered as a whole and the various authorities brought into closer co-operation, and possess the support of one central body as responsible for the health of the nation.

Mr. Hayes Fisher urged that as the Local Government Board had control of most health matters and had touch with all local authorities, its claim to be the central authority was great.

In reply, Lord Rhondda said he had hoped that he would have been able to give definite information on some of the points raised, but unfortunately he was not able to do so at that moment. The Government was seriously considering the question of a Ministry of Health, and in such circumstances —as they would readily understand—he would have to be very careful as to what he said. Even if he gave his personal views, they would not be of great value, but in that respect he must be guarded. Still, they knew his views sufficiently to realise that he was heartily in favour of some central authority dealing with all the health activities of various Departments. In respect of housing, that Department, the Local Government Board, was fully aware of the very great importance and urgency of the matter. He regarded it as the biggest question that they had to deal with, and it must be dealt with soon. It was, however, difficult and complicated. He had been going into it a good deal, and the more he got into the question the more difficult it appeared to him to become. But, however difficult it was, it was a question which the State had to deal with and to take in hand at once. They must take it in hand now if they were to begin after the war without any delay. During the war, owing to financial restrictions and labour difficulties, very little could be done. The problem had become more urgent since the war, and they were fully alive to the close bearing which it had upon health matters. High mortality was undeniably associated with bad housing conditions. Upon the point regarding the compulsory transfer of patients to sanatoria, he had had a communication from medical men pointing out that that was not necessary.

Regarding the Ministry of Health, he had hoped to have been in a position to have gone into details; but they would understand his position. He hoped before long that the Government would come to a decision. All who had given intelligent thought to the question were agreed as to the necessity of a central authority. It resolved itself into a problem as to when it should be taken in hand and in what way it should be done. He could give the deputation the assurance that his plan was to build up and construct on old organisations rather than to dispense with them, and without being too definite, he could say that he was not likely to dispense with the Insurance Committees, whose full co-operation he relied upon. He also wanted the fullest co-operation of the approved societies, and, indeed, of all who were interested in health work. There was no reason to assume that Insurance Committees were going to be abolished. He agreed, too, that the taint of pauperism would have to be completely eliminated, but, regarding the suggestion for the inclusion of non-insured persons in treatment schemes, he was not at the moment prepared to agree to that, though no doubt that even would come in time.

